# The Influence of Physical Exercise Frequency and Intensity on Individual Entrepreneurial Behavior: Evidence from China

**DOI:** 10.3390/ijerph191912383

**Published:** 2022-09-28

**Authors:** Dewen Liu, Shenghao Han, Chunyang Zhou

**Affiliations:** 1 School of Management, Nanjing University of Posts and Telecommunications, Nanjing 210003, China; 2 College of Business, Shanghai University of Finance and Economics, Shanghai 200433, China

**Keywords:** entrepreneurial behavior, physical exercise, exercise frequency, exercise intensity

## Abstract

Physical exercise can benefit individuals’ physical and mental health and also influence individuals’ long-term behavioral choices. Doing exercise is particularly important given that physical exercise can impact individuals’ cognitive abilities and positive emotional states, which may further impact entrepreneurial behavior. Therefore, understanding the relationship between exercise and entrepreneurial behavior is essential, because it can provide policy suggestions for popularizing athletic activities and boosting entrepreneurship. Consequently, the present study examined whether physical exercise could predict entrepreneurial behavior and the possible psychological mechanisms within this relationship. Based on the 2017 Chinese General Social Survey (CGSS2017), this study tested the hypotheses using the Probit and Tobit models. The results showed that individuals’ physical exercise intensity and frequency positively affected their entrepreneurial behavior. In addition, five variables moderated the relationships between physical exercise and individual entrepreneurial behavior: urban–rural differences, education level, marital status, the existence of minor children, and age. Moreover, positive emotions and physical/mental health mediated the influence of physical exercise (exercise frequency and exercise intensity) on individual entrepreneurial behavior. Endogeneity explanations were ruled out by including instrumental variable, copula terms and adopting coarsened exact matching.

## 1. Introduction

Physical exercise is beneficial to individuals’ biological and psychological health [1]. Thus, studies related to physical exercise have long been the focus of scholars in various disciplines. A recent research paper published in *Nature* showed that physical exercise can suppress food intake and obesity [2], underscoring exercise’s fundamental role in deciding individuals’ health. In a similar vein, physical exercise was also proven to be highly correlated with lower risk of cardiovascular morbidity [3], stable blood pressure and heart rate [4], lower overall fatigue [5], functional respiratory parameters [6], and higher self-perceived health status [7]. Studies also indicated that increased exercise participation is critical during the COVID-19 crisis, as it is a therapeutic tool to help individuals overcome difficulties (e.g., [8,9]). In addition to the positive effects on people’s biological functions and well-being, another stream of research also probed that physical exercise benefits people’s mental health [10,11]. Based on a sample of firefighters, Soteriades et al. [12] argued that exercise allows people to experience less occupational stress. This health-promoting effect has been shown in other groups (e.g., college students [13]). Exercise improves people’s cognition functions as well [14,15], such as memory abilities, the efficiency of attentional processes, and executive-control processes [16,17], as exercise is linked with healthy brain development [18]. Exercise also serves as the “interceptor” to prevent people from engaging in unhealthy behaviors (e.g., smoking) [19,20,21]. However, most previous behavioral outcome effects of physical exercise have mainly focused on the immediate body index (e.g., blood pressure and heart rate [4]) or medium-term performance (e.g., academic performance [22]). There is a lack of further inquiry into how physical exercise shapes longer-term behavioral outcomes.

Meanwhile, entrepreneurship has received increased attention in recent years, as entrepreneurship can be an effective way to boost economic growth. According to the Global Entrepreneurship Monitor (GEM) 2020/2021, people’s propensity to start a new business continues to increase in 43 economies worldwide, despite the negative impacts of the COVID-19 pandemic on economic growth. Especially after the epidemic, many economies have fallen into weak growth, and entrepreneurship for employment has become a policy direction for many developing countries. Entrepreneurship has been the highest priority, given the importance of entrepreneurship in boosting economic growth and personal well-being in many regions. For example, the Chinese government entailed a series of policies to encourage people to engage in innovation and entrepreneurship. Studies have focused on how to spark entrepreneurial behavior [23]. Existing studies have found that people’s self-efficacy [24], propensity to take risks [25], personal upbringing [26], and cognitive characteristics [27] can improve their tendencies to engage in entrepreneurial behavior. As an act with long-term orientation and risk of failure, entrepreneurial behavior requires individuals with good physical conditions [28], emotional competence [29], self-management skills [30], and perseverance [31]. In the current literature, physical exercise also correlated with hardiness, a personality style that enables a person to withstand or cope with stressful situations [32]. Accordingly, among numerous antecedent factors related to individual entrepreneurial choice, although physical exercise has been shown to have a “healthy” impact on individuals’ physiological, psychological, and emotional states, its role in personal entrepreneurial choice has been overlooked.

The present study first tried to link physical exercise (exercise intensity and frequency) and individual entrepreneurial choice, to solve the mentioned research gap. This study proposed that exercise intensity will positively predict an individual’s entrepreneurial choice, because people who do exercise with a high level of intensity will have more self-efficacy and courage [33,34,35], which is an important driving force for starting their own business [24,25]. Exercise frequency facilitates an individual’s entrepreneurial choice, because launching a business has been regarded as an enormous pressure, and regular exercise helps people deal with the stress of entrepreneurship [12,36,37]. Second, this study examined and compared five contingency factors (e.g., urban–rural differences, education level, marital status, the existence of minor children, and age) to explain when the positive effect of exercise on entrepreneurial choice would be more evident and significant. Investigating moderators (heterogeneity) is essential, as Zhang et al. [38] pointed out that there was high variability in the impacts of physical exercise on individuals’ social behaviors. Third, this study sought to identify the two important mediators (e.g., positive emotions and physical/mental health) in bridging physical exercise and entrepreneurial behavior. Exploring the mediating mechanism is also essential, as it can better make sense of the internal process in the relationship between physical exercise and entrepreneurial behavior. Fourth, to ensure the robustness of the results, this study used various methods, including instrumental variables, coarsened exact matching, reduced sample re-estimation, and copulas (an instrument-free method), to exclude endogeneity explanations. This study used the samples from the 2017 Chinese General Social Survey (2017CGSS), national-level data from by the National Survey Research Center of China, to examine the hypotheses, guaranteeing the validity of this study. The objective of the current study was to examine the different dimensions of exercise among entrepreneurs and non-entrepreneurs as well as explore the potential association between exercise and entrepreneurial behavior among Chinese citizens. 

## 2. Literature Review

### 2.1. Entrepreneurial Behavior

Entrepreneurship is the process by which individuals optimize and integrate the resources they own to create more excellent economic or social value. In fast-moving business environments open to global competition, entrepreneurship is an effective way to promote innovation and attract invention [39,40]. Therefore, many studies began to explore the antecedent factors of entrepreneurship [41]. Existing studies have focused on individual endogenous differences in people’s entrepreneurial behavior [42,43,44,45]. A more detailed description can be found in Ref. [46], which systematically reviewed several meta-analytical works about the impacts of individual differences on entrepreneurial choice. Among many individual differences that can impact entrepreneurial behavior, an individual’s self-efficacy is the strongest predictor of entrepreneurial behavior [47]. Self-efficacy is essential for entrepreneurs, as they must be assertive in their abilities to accomplish various tasks in uncertain conditions [48]. Individuals with high self-efficacy are likely to persevere when problems arise and search for challenges, seizing the opportunity of entrepreneurship [49]. Beyond this point, other traits, such as risk propensity and stress tolerance, also positively correlate to individual entrepreneurial choice [47,50]. Since entrepreneurs make various decisions in uncertain situations, which will lead to significant risk and pressure [51,52,53], entrepreneurs need a strong ability for stress tolerance. Additionally, people with a high ability in self-management are more likely to initiate entrepreneurship, as they can better manage time and various unanticipated issues during the entrepreneurship process [54].

Although the past literature has generated many insightful contributions on identifying the antecedents of entrepreneurship from an endogenous perspective, existing studies have failed to explore how to enhance individual entrepreneurial propensity from an exogenous perspective, which remains a gap that this study tries to fill. Indeed, beyond the role of individual endogenous differences, research has also proposed that individuals’ past experiences also influence entrepreneurial choice [55]. In other words, entrepreneurship is not innate, but environmental variables may also affect the choice of entrepreneurship. Therefore, it is reasonable to assume the impacts of individuals’ experiences and habits (e.g., physical exercise) on the choice to start a business or not. It is also worth mentioning that entrepreneurship is usually associated with high risk and pressure [39,56]. Therefore, entrepreneurial individuals need to have greater tolerance toward risks and stress. In this regard, physical exercise may be an essential tool for starting entrepreneurship, as a necessary means of relieving pressure.

### 2.2. Positive Aspects of Physical Exercise

Sports science and health researchers have spent many efforts investigating how physical activity impacts individuals from multiple perspectives, including physical and mental health, cognitive response, and so on.

Exercise benefits people’s physical and mental health. Epidemiological studies showed that moderately active individuals are at a lower risk of mental illness than sedentary individuals, indicating that physical exercise promotes an individual’s physical and psychological development [57,58,59]. Physical exercise was also being found to have a robust antidepressant effect [38]. This stream of research suggested that physical exercise has a positive effect on individual cognitive functions [44]. Physical exercise also significantly enhances memory performance [60,61]. It is evident that this effect is mainly through changing the brain structure and increasing the function of neurotransmitter levels, to improve individuals’ immediate cognitive performance [18,62].

Scholars have also found that physical exercise affects individuals’ actual behaviors. For example, one study demonstrated that physical exercise could enhance people’s creativity [63], and this effect also applied to East Asian populations in Hong Kong [64]. Physical exercise also prevents people from smoking, drinking, gambling, and overeating [19,20,21], leading them to develop a healthy lifestyle.

More relevant to this study, in the management field, there are studies investigating the relationship between physical exercise and individual behaviors in the workplace. For example, the management literature suggests that physical exercise can enhance an individual’s work performance [61]. Physical activity among these business owners positively predicts business performance [65]. Exercise can help people improve work-related self-efficacy, while reducing ego depletion [66,67]. Given that there are many stressful events in the workplace [68,69,70], research has proven that physical exercise could be an effective tool to deal with various pressures and stress [71,72,73,74,75]. It is worth noting, however, that the existing studies failed to examine the long-term behavioral outcomes of physical exercise (e.g., entrepreneurship), making it impossible to link physical exercise with entrepreneurial behavior.

## 3. Hypotheses Development

Exercise may involve different aspects, dimensions, and purposes. For example, specific physical exercise aims at force, resistance, coordination, mobility, or speed. However, for average residents, their daily physical exercise may not be divided in such a detailed manner. Especially for Chinese respondents, the research sample of this study, a national fitness program has just been proposed, so Chinese people’s physical exercise is only to strengthen their bodies. Thus, in this section, this study focuses on two dimensions (exercise intensity and exercise frequency) of physical exercise and presents how exercise intensity and frequency impact an individual’s entrepreneurial choice. Exercise intensity is defined as how hard people work during exercise, usually reflected in a high level of heart rate and oxygen consumption [76,77,78]. Exercise frequency is defined as how often people exercise [79]. In general, exercise intensity is usually more strongly associated with an individual’s cardiorespiratory capacity [80], while exercise frequency is more strongly associated with competences in self-regulation and self-management [81].

This study first argued that exercise intensity positively affects an individual’s entrepreneurial choice. People may face significant uncertainties when deciding to launch a business [24,25]. Compared with stable work, launching a business requires people to care about the uncertain business environment and unstable income. Given that uncertainty and instability accompany an individual’s entrepreneurial choice, this paper inferred that those who are self-confident and brave are more likely to take risks rather than live a stable and unchanging life [82,83]. Studies suggested that a high level of exercise intensity brings self-efficacy and courage to people, as people may feel pride when they successfully engage in physical exercise with a high level of exercise intensity [33,34,35], which enables individuals to have the ability, motivation, and willingness to perform challenging works. This is true for people to make an entrepreneurial choice, as they will usually confront many uncertainties and challenges [40].

Then, this study proposed that exercise frequency is positively related to an individual’s entrepreneurial choice. Management researchers endorsed that regular exercise is an effective way to cope with work-related stress [66,67,68,69]. When considering being an entrepreneur, people should assume the role of decision-maker, a position that can come with a lot of pressure and loneliness. Many individuals cannot share or confide the possible difficulties during entrepreneurship with friends and family members, and this pressure can manifest itself in emotional eating, low sleep quality, and poor mental health [51,52], preventing people from choosing entrepreneurship. In this sense, physical exercise is likely to promote entrepreneurship, as it relieves stress and promotes cognitive functions [12,36,37]. In addition, research also found that self-disciplined individuals are more likely to choose entrepreneurship [84]. Accordingly, those who exercise regularly are more disciplined, resulting in an inclination for choosing entrepreneurship.

In addition, both exercise intensity and frequency improve people’s social skills and social capital [85,86]. During constant social interactions, individuals who frequently engage in physical activities are more likely to display confidence, energy, charm, vigor, and extraversion. Thus, they will have more chances to leave a stunning impression, contributing to their social network ability [87]. Further, Zhang et al. [38] found that physical exercise improves individuals’ communication skills, adding new ways for individuals to interact with others and heterogeneity to their social networks and, ultimately, enhancing their social capital, an essential predictor of entrepreneurship [88].

To sum up, this study believed that individuals who exercise regularly might be more likely to engage in entrepreneurial choice than those who do not exercise regularly. This paper then examined whether exercise intensity and frequency affect the choice of entrepreneurial behavior, the analysis of moderating factors, and the possible mediating transmission mechanisms. Thus, the following hypotheses were proposed:

**Hypothesis** **1** **(H1):** *Physical exercise intensity positively relates to an individual’s entrepreneurial behavior*.

**Hypothesis** **2** **(H2):** *Physical exercise frequency positively relates to an individual’s entrepreneurial behavior*.

## 4. Methodology

### 4.1. Research Data

This study obtained the data from the 2017 Chinese General Social Survey (CGSS2017). CGSS is China’s national, comprehensive, and continuous large-scale social survey project. The 2017 version of the CGSS questionnaire used a random sampling method to survey residents in various regions of China, and a final valid sample of 12,582 was obtained. The survey was distributed during summer vacation (July and August) of 2017, and all variables were collected during this period. After removing the samples that did not fit this study (missing key variables or refusal to answer), the final sample included in this paper was more than 9800.

### 4.2. Variables

The dependent variable in this study is entrepreneurial behavior. Referring to other studies on entrepreneurial behavior based on this national survey [89], this study determined whether the respondents’ current work status is “owner or partner, self-employed”, to assess whether they make an entrepreneurial choice or not.

The independent variable in this study is physical exercise. Referring to the other literature on physical exercise based on the 2017 version of the CGSS questionnaire [90], two self-reported questions about physical exercise ask participants to answer the intensity and frequency of physical exercise, respectively. The former fill-in-the-blank question asked, “In the past 12 months, how many times a week did you engage in physical exercise that lasted 30 min and made you sweat?”, while the latter question asked respondents to self-assess the frequency of physical exercise in the past year (never, several times a year or less, several times a month, several times a week, and every day). The correlation between these two variables was 0.443 (*p* < 0.01), showing adequate correlation.

The selection of control variables is required to ensure accurate study results. The authors selected the control variables in the following two ways. On the one hand, this study searched for studies related to entrepreneurship. The relevant conventional variables were included as control variables. For example, Santos et al. [31] included gender, age, socioeconomic status, and education as control variables in studying the relationship between personal values and entrepreneurial behavior, as these variables were proven to affect individuals’ entrepreneurial behavior significantly. Xiao and Wu [91] and Bates et al. [92] underscored the importance of marriage and ethnic minority in entrepreneurship. The current study also showed that the number of kids and household registration also influence individuals’ entrepreneurship [93,94].

On the other hand, the authors retrieved the literature that examined individual entrepreneurial behavior based on CGSS data. For example, Liu and Yang [95] further included political status and place of residence in their study, as the two are essential in social mobility in China. Moreover, Hafer and Jones [96] believed that individual cognitive abilities should be further controlled when studying entrepreneurship. Based on the above, this study adopted a similar approach to these previous studies and included the following relevant control variables: (1) gender; (2) region (western, central, and eastern); (3) age; (4) whether an ethnic minority; (5) marital status (married, including married and remarried; unmarried including single, divorced, widowed, and cohabiting); (6) political status (CCP or non-CCP members); (7) education (illiterate, private school/literacy class/elementary school, junior high school, high school/technical school/junior high school, high school, undergraduate, and postgraduate and above); (8) annual personal income; (9) number of underage children; (10) whether agricultural household registration; (11) individual’s cognitive ability (listening and speaking level of Mandarin/English); (12) whether owning properties (e.g., commodity housing); and (13) place of residence (rural, township/market town, prefecture-level city/county, and municipality/provincial capital city). Table 1 presented the descriptive statistics (means and standard deviations, SD) of all variables for the entire sample, both non-entrepreneurial and entrepreneurial groups. Most variables differed significantly between two groups, which later laid the foundation for further analysis. In addition, the Harman single factor test results showed that the first factor could explain 22.91% of the variance, and the cumulative factors can explain 63.63% of the variance. The variance explained by the first factor was less than 50% or half of the total variance, indicating that the common method bias was not severe in this study [97].

## 5. Results

The main objective of the formal analysis was to examine the direction and magnitude of the effect of physical exercise intensity and frequency on entrepreneurial behavior. Thus, this study first treated these two variables as independent variables and the entrepreneurial behavior as the dependent variable in Models 1–4. Further, this study continued to explore the group differences in the effect of physical exercise on entrepreneurial behavior; that is, whether there is a heterogeneous effect of physical exercise on entrepreneurial behavior across various groups. This study chose to examine the difference of urban/rural, educational background, marital status, existence or not of minor children, and age (young and non-young), and the results can be seen in Models 5–36.

### 5.1. Main Effects

Given that the dependent variable was binary (0 = non-entrepreneurship; 1 = entrepreneurship) in this study, and the large proportion of the sample belongs to non-entrepreneurship (nearly 90%), this study used the Probit and Tobit models to test the main effects. Previous research argued that if the discrete choice model contains some endogenous explanatory variables, it is theoretically easier to deduce through the Probit model compared to other contest models (e.g., the Logit model), as the model denotes the probability. The Probit model has been applied in many similar entrepreneurial studies, due to its advantages in explaining the probability of behavioral outcomes (e.g., [98]). Moreover, the dependent variable is roughly continuously distributed on the positive value but contains a part of the observations with a positive probability value of zero, which is also suitable for the Tobit model. Model 1 in Table 2 showed that physical exercise intensity positively correlated with entrepreneurial behavior (b = 0.008, *p* < 0.05). This result indicated that the probability that physical exercise contributed to increased entrepreneurship is 0.008, corresponding to 9.41% of the sample mean (i.e., 0.008/0.085).

Similarly, Model 2 in Table 2 showed that physical exercise intensity was positively related to entrepreneurial behavior (b = 0.028, *p* < 0.05), indicating that the probability of choosing entrepreneurial behavior increases by 0.028 for each increase in the frequency of physical exercise, which corresponds to 32.94% of the sample mean. The Tobit coefficient estimated the linear increase in the latent variable for each unit increase in the predictor. Thus, the study used the Tobit model to re-estimate, and the regression coefficients of exercise intensity and exercise frequency in Table 2 were 0.012 (*p* < 0.01, Model 3) and 0.042 (*p* < 0.05, Model 4), respectively, indicating adequate positive impacts of exercise intensity and frequency on entrepreneurial behavior and the robustness of the results. Taken together, the two estimation methods showed that active exercise increases the probability of individuals engaging in entrepreneurial behavior jointly, supporting H1 and H2.

The regression coefficients showed that exercise frequency has a greater positive effect on entrepreneurial behavior. The present study hypothesized that this might be due to the increased frequency of physical exercise representing the regular physical activity of individuals over a relatively long period, which improves their quality of life and their intention to challenge themselves [99,100]. In our research sample, the highest frequency was to exercise every day, which is an acceptable level of exercise. In contrast, exercise intensity had a more limited positive effect on individual entrepreneurial behavior. This may be since prolonged commitment to high-intensity exercise has a limited marginal effect [101]. Thus, this paper argued that regular exercise is more likely to help individuals obtain positive results from exercise.

Regarding the control variables in the regressions, entrepreneurial behavior did not significantly differ between the western and eastern regions, the presence or absence of ethnic minorities, and different resident places, which showed that entrepreneurship is a national “cause” in China, and many ordinary people are engaged in it. It is interesting to note that education and individual cognitive ability harmed entrepreneurship. This may be because China’s economy was running well before 2018, and most residents with high educational backgrounds and cognitive ability tended to choose employment rather than start their own businesses. Besides, political appearance negatively influenced entrepreneurial behavior. In other words, communists are more reluctant to undertake entrepreneurial ventures. This study argued that by being in a country where the Communist Party is the ruling party, China’s CCP members might prefer to receive fixed payments within the system, as opposed to the additional risk of entrepreneurship.

### 5.2. Urban–Rural Difference

Given that the Probit model performed better than the Tobit model in the above analysis, this study then used the Probit model to conduct the heterogeneity analyses in the following analysis. In China’s dualistic urban–rural system, there may be significant differences between the influence of physical exercise on entrepreneurial behavior. There is a massive gap between the geographical structure, living environment, and even further entrepreneurial ecology in rural and urban areas. Therefore, this subsection will separately examine the relationship between physical exercise and the entrepreneurial choice of rural and urban residents.

As shown in Table 3, both exercise intensity (b = 0.012, *p* < 0.05, Model 5) and exercise frequency (b = 0.067, *p* < 0.01, Model 6) significantly and positively affect the individual entrepreneurial behavior of rural residents. In contrast, this effect was not significant for urban residents, which implies that the effect of regular exercise may be more significant for rural residents. Three reasons may explain this finding: (1) Rural residents do not have better physical exercise facilities than urban residents. China has invested lots of funds in the construction of exercise facilities in urban areas, while under-investing in rural areas. Therefore, the quality of physical exercise performed by residents in rural areas may be relatively low. Thus, the rural residents who exercise regularly will obtain a more significant marginal effect. (2) The positive effect of exercise on entrepreneurial behavior for urban residents is not evident, because urban residents have more abundant sports resources available. For example, they can go exercise in the community or go to gyms to receive professional exercise instructions. Thus, urban residents are less appreciative of the opportunities to exercise, diminishing the positive effects of exercise. (3) For urban dwellers, cities have more abundant employment opportunities, which also weakens the positive impact of a single factor on entrepreneurship. The coefficients of interaction terms were all statistically significant (b = −0.013, *p* < 0.05, Model 7 and b = −0.118, *p* < 0.01, Model 10), suggesting that being an urban resident negatively moderated the effect of exercise on entrepreneurship.

### 5.3. Educational Background Difference

Table 4 further examined whether educational background moderated the effect of physical exercise on the choice to start their businesses. For residents with a low education level (junior high school and below) and residents with a medium education level (high school), the effect of exercise intensity on entrepreneurial behavior was not significant. In contrast, exercise intensity positively affected entrepreneurial behavior (b = 0.019, *p* < 0.01, Model 13) for residents with a high education level (graduate degree or above).

However, the opposite result was observed when physical exercise frequency was used as the independent variable. The positive effect of exercise frequency on entrepreneurial behavior was only found in the low-education-level group (b = 0.035, *p* < 0.05), rather than in the medium- or high-education-level groups. There were two potential reasons: (1) Residents with a low education level have less time and fewer opportunities to exercise, as they have to devote more time to make a living. In China, less educated individuals often engage in heavy or repetitive work and may also need to work multiple jobs to make a living. Starting a business may become a decision that changes this status quo. So, the positive effect of exercise frequency became more prominent, as regular exercise provides them essential ways to relax their minds and bring them out of their “boring” lives. (2) Residents with a high education level have long been engaged in brain-oriented work. They are often engaged in work that requires creativity, experiencing tremendous mental stress. Thus, they need more intense physical exercise to relieve themselves from the difficulties and stress in their workplace than light regular physical exercise. Thus, exercise intensity plays a more significant role in promoting their courage and willingness to initiate a business. Moreover, the interaction terms between exercise intensity (b = 0.004, *p* < 0.1, Model 14)/exercise frequency (b = −0.021, *p* < 0.05, Model 18) and education positively/negatively predicted the outcome of entrepreneurial behavior; that is, education played a moderating role in the relationship between exercise and entrepreneurial behavior.

### 5.4. Marital Status Difference

The analysis in this subsection aimed to determine whether marital status significantly moderated the relationship between physical exercise and entrepreneurial behavior. Given that an essential physiologic function of physical exercise is to alleviate stress, married residents may face tremendous pressure, mainly from the family in their lives, compared to unmarried residents. For example, married individuals often need to deal with multiple conflicts between family and work. Therefore, this study further examined the differential effect of physical exercise on entrepreneurial behavior between the married and unmarried population in Table 5. As anticipated, both exercise intensity (b = 0.009, *p* < 0.01, Model 20) and frequency (b = 0.027, *p* < 0.1, Model 23) positively influence the entrepreneurial behavior of married people. The possible reasons are as follows: (1) Marriage may give individuals a new social role (e.g., a spouse) and place social pressure on them, so, whether it is regular or intense, exercise is an effective means of relieving the stress in their lives and restoring their “energy”; thus, the positive effect of physical exercise will be more prominent for them. (2) Under the “control” of the family, married people may live more regular lives to coordinate with their family members’ pace, which allows for a synergistic effect of exercise and regularity to guide individuals to cope with challenges adequately. Therefore, for married people, the positive spillover effect of physical activity becomes more influential, making these individuals more likely to engage in entrepreneurship. However, the interaction terms in Model 21 (b = 0.013, NS) and Model 24 (b = −0.016, NS) were insignificant, implying no significant marital difference in promoting entrepreneurship through exercise.

### 5.5. Underage Kids Difference

This study further examined whether there is a difference in the utility of physical exercise for those with and without underage kids. The logic of this subsection was similar to that of Section 5.4, because having minor children also stresses the lives of individuals who are parents. As shown in Table 6, the utility of exercise was more remarkable for residents with underage kids. The positive effects of exercise intensity (b = 0.012, *p* < 0.05, Model 26) and frequency (b = 0.054, *p* < 0.05, Model 29) on entrepreneurial behavior were only significant for individuals with underage kids. This paper argued that raising underage children means that parents must devote their time and energy to the companionship and nurturing of their underage children, especially in China, where Confucianism and collectivism are prevalent. Like the marital situation (Section 5.4), exercise can somewhat alleviate the stress in this process and help individuals establish confidence in dealing with difficulties.

Further interaction results showed that the number of minor children moderates the positive effect of exercise frequency (b = 0.049, *p* < 0.01, Model 30) on entrepreneurial behavior but not the positive effect of exercise intensity (b = 0.007, NS, Model 27) on entrepreneurial behavior. This paper argued that this finding is due to the constant stress of raising underage children, which requires regular exercise to alleviate the stress.

### 5.6. Age Difference

A difference existed in the role that exercise played at different stages of an individual’s life. For example, an individual may lack vital physical functions in their elder years, resulting in diminished gain from exercise. According to the World Health Organization (WHO)’s classification and sample characteristics, this study chose age 44 as the cut-off point to divide young and non-young groups [102]. This paper argued that young people are at a prime stage of life, when exercise has a greater contribution to their improved cardiorespiratory fitness and physical performance, which may allow exercise to be more effective. This test aimed to investigate whether there were age differences in the effect of physical exercise on entrepreneurial behavior.

As shown in Table 7, exercise intensity (b = 0.010, *p* < 0.01, Model 31) and exercise frequency (b = 0.052, *p* < 0.01, Model 34) only exerted positive effects on entrepreneurial behavior significantly for individuals who were younger than 45 years old, indicating that young people will gain more from exercise. However, this positive effect was not significant in the non-young-adult group. The interaction terms were significant in Model 36 (b = −0.002, *p* < 0.01) but insignificant in Model 33 (b = 0.000, NS). Thus, age only moderated the relationship between exercise frequency and entrepreneurial behavior. 

## 6. Further Analysis

In the above analysis, this study demonstrated the relationship and between-group differences in the effect of physical exercise on entrepreneurial behavior. In general, physical exercise can positively promote an individual’s entrepreneurial behavior. However, it remained unclear how and why physical exercise facilitates an individual’s entrepreneurial behavior. Thus, the primary purpose of the further analysis was to test why physical activity could positively promote individuals’ choice of entrepreneurship, based on the available data in the 2017CGSS. The results can be seen in Section 6.1. Further, given the possible endogeneity issues in this study (e.g., omitted variables or inverted causality), in Section 6.2, this study further employed various methods to rule out endogeneity to demonstrate the results’ robustness.

### 6.1. Mediating Tests

This study argued that physical exercise might ultimately influence individuals’ entrepreneurial behavior through three important bridging mediators. (1) Positive emotions: physical exercise can promote dopamine secretion, enabling individuals to maintain a positive attitude toward life, thus contributing to a greater preference for starting a business. (2) Physical and mental health: physical exercise can help individuals maintain a good physical and mental state, thus enabling them to cope with various challenges. Respondents in the 2017 version of the CGSS questionnaire reported their subjective positive emotions, height, weight, and subjective health perception, so this study used one indicator (subjective positive emotion) to measure positive emotions and two indicators (BMI and subjective perception of physical and mental health) to measure physical and mental health.

According to the intermediary test method proposed by Zhao et al. (2010) [103], the results are presented in Table 8 and Table 9. As shown in Table 8, the positive effect of exercise intensity on positive emotions was significant in Model 37 (b = 0.006, *p* < 0.05), and the positive effect of exercise intensity on entrepreneurial behavior remained significant in Model 38, when positive emotions were included in the regression equation (b = 0.014, *p* < 0.01). Accordingly, this study inferred a mediating effect of positive emotions in the relationship between exercise intensity and entrepreneurial behavior. In a similar vein, physical and mental health played a mediating role between exercise intensity and entrepreneurial behavior. However, in Model 39, the effect of exercise intensity on an individual’s BMI is insignificant (b = 0.008, NS), and a further Sobel test showed that the mediating effect of BMI was not significant (Z = 0.972, *p* > 0.05). This study inferred that BMI, as a physical health indicator, did not mediate the relationship between exercise intensity and entrepreneurial behavior. This finding is consistent with previous research findings that aerobic exercise, instead of anaerobic exercise, has a more significant role in improving physical health [104]. That is, exercise intensity was usually independent of BMI in this study.

As shown in Table 9, the positive effect of exercise frequency on positive emotion was significant in Model 43 (b = 0.043, *p* < 0.01), and the positive effect of exercise frequency on entrepreneurial behavior was not significant when positive emotion was included in the regression equation (b = 0.035, NS, Model 44), indicating that the mediating effect of positive emotions between exercise frequency and entrepreneurial behavior was not significant. Similarly, the mediating effect of physical and mental health between exercise frequency and entrepreneurial behavior was insignificant; however, in Model 45, exercise frequency positively related to an individual’s BMI (b = 0.099, *p* < 0.01), and, when BMI was included in the regression equation, the positive effect of exercise frequency on entrepreneurial behavior remained significant (b = 0.025, *p* < 0.1). Therefore, the mediating effect of BMI (i.e., physical fitness) between exercise frequency and entrepreneurial behavior was significant. It should be noted that although excessive BMI (≥28) represents obesity, only a tiny proportion of the sample in this study had a BMI in the overweight range. Therefore, the analysis of BMI’s mediating role was still relevant. This paper argued that the mediating effect of positive emotions and physical and mental health was insignificant because the emotional and cognitive enhancement induced by exercise frequency is diluted in terms of the long-term effect. An individual’s emotional and psychological state will stabilize over time. Therefore, this paper found that the entrepreneurial behavior triggered by exercise frequency was mainly determined by shaping the physical qualities of individuals.

### 6.2. Robustness Checks

Although the above analysis revealed that physical exercise was strongly associated with entrepreneurial behavior, it did not comprehensively explain a causal relationship between physical exercise and entrepreneurial behavior. When analyzing the effect of physical exercise on entrepreneurial choice using large-scale survey-type data, the critical point is to control for selection bias and the endogeneity caused by omitted variables and reversed causality. Whether or not to engage in entrepreneurship is an individual’s personal choice, and both observable variables (e.g., gender, age) and unobservable ones (i.e., entrepreneurship support) may influence this choice. If this endogeneity is not corrected, it can create problems of overall bias in the results.

This study adopted the following approaches to exclude endogeneity explanations: instrumental variable, coarsened exact matching, reduced sample analysis, and copulas. This study first adopted the instrumental variable method, which requires that the instrumental variable is highly correlated with the independent variable but unrelated to the dependent variable. Therefore, the amount of time spent watching live sports matches in the 2017 version of the CGSS questionnaire was used as the instrumental variable. Respondents’ viewing of live sports matches reflected their enthusiasm for physical activities and inevitably affected their frequency and intensity of participation in physical exercise, but this variable does not affect their actual choice of occupational behavior. The regression result of the instrumental variables approach was presented in Table 10. After excluding endogeneity, the effects of physical exercise intensity (b = 0.140, *p* < 0.05, Model 51) and exercise frequency (b = 0.207, *p* < 0.05, Model 52) on entrepreneurial behavior remained significantly positive. It should be noted that the weak instrumental variable tests (Wald test and AR test) for both models showed that the instrumental variable used in the study did not have a weak instrumental variable problem and was suitable for this study.

Further, this study used coarsened exact matching (CEM) to control the effect of confounding factors on entrepreneurial behavior [105]. CEM is a method to improve the estimation of causal effects by reducing the imbalance of covariates between the control and treatment groups. Compared with other traditional methods (e.g., propensity score matching (PSM)), CEM has the following advantages: (1) CEM satisfies the principle of consistency. CEM can match directly based on the empirical distribution of the original data, rather than needing to be matched based on the common region of the two groups of data. (2) CEM is able to preserve the original data as much as possible, since the sample size of the two groups of data after CEM need not be equal, while traditional methods require equal numbers for the two groups of data. (3) CEM conducts matching based on the theoretical antecedents between variables, thus reducing the dependence on the model. (4) CEM is more convenient and effective when processing large samples.

The general steps of CEM are as follows: (1) Covariates are stratified according to user-defined split points. In this study, respondents’ age, marital status, being a CCP member or not, education, total income, number of underage children, and cognitive ability were selected for stratification, because these variables all have significant and positive effects on whether or not to start a business, and differences in these covariates may affect the estimated effects of physical exercise. (2) Using an exact matching algorithm, strata were created based on the empirical distribution of the sample, and subjects in each stratum (those containing at least one intervention group and one control group) were matched precisely; otherwise, the stratum was removed. (3) The successfully matched subjects were retained, and the matched data were used to test the relationship between physical exercise and entrepreneurial behavior. Considering CEM to select specific treatment and control groups for this study, exercise intensity and frequency were transformed into dummy variables by assigning 0 to people who had never exercised and 1 to people who had experienced exercise. The specific CEM results can be found in Table 11. It can be observed that the estimated coefficients of exercise intensify (b = 0.010, *p* < 0.01, Model 53) and frequency (b = 0.033, *p* < 0.05, Model 54) were significantly positive.

This study also selected respondents between 18 and 45 to test again. The reason for this is that, on the one hand, individuals who have reached the age of 18 have reached adulthood in the legal sense and have the full civil capacity to choose entrepreneurial activities; on the other hand, for most people, their careers gradually enter a late stage after the age of 45, so individuals are less likely to continue entrepreneurial activities. The specific test results are shown in Model 55 and Model 56 of Table 11, consistent with the findings in Section 5.5. To further eliminate the concern about reserve causality, this study employed the instrument-free method introduced by Park and Gupta (2012), using Gaussian copulas [106]. Copulas are a class of functions that allow the joint distribution of endogenous variables and the error term to be constructed from the individual marginal distributions, thereby accounting for the correlation between them. As Park and Gupta (2012) suggested, the copula method provides unique advantages over the instrumental variable approach, which often suffers from validity and weak instrument issues [106]. The instrument-free method has been increasingly adopted in previous research (e.g., [107]). Following Park and Gupta (2012)’s suggestion [106], this study estimated the empirical distribution of each focal variable nonparametrically using an Epanechnikov kernel density function and calculated the bandwidth. Then, the copula terms were added in the regressions as covariates. The results are shown in Model 57 and Model 58 of Table 11. It can be observed that the copula terms are insignificant, indicating no serious reverse causality problem in this study. The focal variables’ regression coefficients (b = 0.008, *p* < 0.1, Model 57; b = 0.055, *p* < 0.05, Model 58) remained significantly positive.

The above robustness results suggested that this study’s findings were robust, in that physical exercise promotes individuals’ choice to engage in entrepreneurship.

## 7. Conclusions

### 7.1. Main Findings

This study tested the relationship between physical exercise and entrepreneurial behavior, based on 2017 Chinese General Social Survey data, and demonstrates that individuals’ physical exercise intensity and frequency positively affect their entrepreneurial behavior. Five variables moderated the relationships between physical exercise and individual entrepreneurial behavior: urban–rural differences, education level, marital status, the existence of underage children, and age. Moreover, positive emotions and physical/mental health mediated the influence of physical exercise (exercise frequency and intensity) on individual entrepreneurial behavior. In addition, this study used various robustness checks (i.e., instrumental variable method, coarsened exact matching, reduced sample analysis, and copulas) to demonstrate the robustness of the results.

Collectively, this paper formed the following findings: (1) Individuals’ intensity and frequency of physical exercise positively affected their entrepreneurial choice, and exercise frequency had a greater effect on entrepreneurial behavior. (2) In the heterogeneity analysis, firstly, both exercise intensity and frequency had significant and positive effects on entrepreneurial behavior for rural but not for urban residents. Secondly, exercise intensity only positively influenced the entrepreneurial behavior of residents with a high education level, and exercise frequency only positively influenced the entrepreneurial behavior of residents with a low education level. Thirdly, exercise intensity and frequency positively contributed more to entrepreneurial behavior for married individuals. Fifth, exercise intensity and frequency positively affected the entrepreneurial behavior of residents with underage children. Finally, exercise only positively affected young individuals (≤44). (3) Exercise intensity indirectly affected entrepreneurial behavior through positive emotions and physical/mental health, and exercise frequency indirectly affected entrepreneurial behavior through physical health. In other words, positive emotion, physical fitness (BMI), and physical/mental health were potential mechanisms of physical exercise’s influence on individual entrepreneurial behavior. This result supported the findings in previous studies, which mentioned that physical exercise helps individuals maintain a positive attitude toward life, improves people’s physical and mental states [108], and, ultimately, improves their entrepreneurial behavior.

### 7.2. Theoretical Contributions

To the best of our knowledge, this is the first attempt to evaluate the association between exercise and entrepreneurial behavior based on a large-scale national survey. It consolidated and organized the limited and scattered literature in public health. Thus, this study contributed to current research in the following way. First, this study drew greater academic connections between physical exercise and the broader literature on entrepreneurial behavior. Prior research almost always focused on the short-term effects of physical exercise, and few studies have further explored the effects of physical exercise on individuals’ long-term behavioral choices. In contrast, studies have shown the linkage between exercise and occupational stress [12], attention [109], anxiety [110], creativity [63], and even subjective well-being [111]. A recent literature review also suggested long-term cognitive improvements in individuals from physical activities (see [112]). Thus, compared to other similar studies, this work goes beyond the current scope of the existing literature by exploring a long-term behavioral consequence, entrepreneurial behavior, echoing the calls for more long-term behavioral outcomes of physical exercise [112]. Besides, this study further identified a new antecedent of entrepreneurial behavior by demonstrating the facilitated role of exercise frequency and intensity, providing a new insight to promote entrepreneurship through lifestyle interventions (e.g., encouraging physical activity) for management studies. Therefore, this paper contributed to the literature in the fields of exercise science and management.

Second, beyond the current research, this study further explored when (moderators or heterogeneity) and why (mediators) physical exercise will influence individual entrepreneurial behavior, thus shaping a more comprehensive understanding of the linkage between physical exercise and entrepreneurial behavior. Third, in terms of methods, this study also fully considers the endogeneity of causal inference in the analysis section, guiding future researchers in demonstrating the causal relationship based on large-scale survey data. Relevant studies have not strictly focused on the causal relationship between the independent and dependent variables, and only a few scholars have deeply explored the effect of physical activity on outcomes, after excluding endogeneity [63]. Under the premise of controlling for endogeneity using the instrumental variable, this paper further adopted the coarsened exact matching approach and copulas to re-match/re-estimate the sample to demonstrate the robustness of the conclusions. Existing studies on physical exercise used a relatively small sample to drive conclusions, such as students or elderly adults via survey or experiment designs, which prevents scholars in this field from acquiring general conclusions. This study’s data analysis is based on 9800 samples from the 2017 Chinese General Social Survey. Thus, the results can be justified on a universal level.

### 7.3. Practical Implications

The above findings have important practical implications for administrations, entrepreneurs, and managers. First, this study inspires how the government promotes entrepreneurship. Compared with other direct approaches to promote individual entrepreneurship (e.g., lowering the threshold of entrepreneurship and taxes for start-ups), this study further suggests a new way for the government to encourage entrepreneurship: promoting physical exercise. Accordingly, this study suggests that administrators and local governments should build sports facilities to facilitate residents’ exercise. In particular, the study found a greater positive effect of physical activities on entrepreneurial behavior in rural areas, illustrating the necessity to establish facilities in rural areas that are suitable for the local population to perform daily exercise. Second, this study provides lessons for individuals who intend to start a business. Entrepreneurs should do more physical exercise, which can help them develop the healthy body and positive emotions they need to start a business, and this positive effect remains significantly positive for high-social-pressure individuals (e.g., married, with kids). Moreover, since exercise frequency positively affects entrepreneurial behavior, this paper suggests that individuals should develop a regular daily exercise program. Third, given the current influx of intrapreneurship in many countries, various successful projects in large companies have been founded by insiders. Our research also suggests that managers might consider adding fitness facilities (e.g., elliptical trainers) in the workplace, so that employees have conditions for physical activity, thus inspiring them to pursue intrapreneurship.

### 7.4. Limitations

Inevitably, this study also has some limitations. First, due to data limitations, this study did not further examine the effects of different types of physical exercise on entrepreneurial behavior. For example, extreme sports may be more likely to motivate individuals to challenge themselves (e.g., entrepreneurship). Second, entrepreneurs comprise only a small portion of almost all the research literature on entrepreneurial behavior, including this paper. Although this study methodologically attempted to address this issue, future attempts can be made to find more balanced data to validate the causal relationships proposed in this study. For example, if there is an opportunity to conduct field experiments, the relationship and causality between physical exercise and individual entrepreneurial behavior can be more rigorously argued. Third, although this paper verified the mechanism of physical exercise on individual entrepreneurial behavior based on the available data, more mediating variables (e.g., personal willpower) can be explored in the future. Finally, the current study used average individuals as research samples. The future questionnaire could address entrepreneurs who have already founded a company and establish how physical exercise contributes to their choice in managing their business.

## Figures and Tables

**Table 1:** Descriptive statistics.

Variables	Full Sample		Entrepreneurship = 0		Entrepreneurship = 1		*t*-Test
	Mean	SD	Mean	SD	Mean	SD	
Exercise intensity	2.330	5.288	2.287	5.020	2.772	7.605	−2.865 ***
Exercise frequency	1.495	1.594	1.480	1.599	1.632	1.537	−2.993 ***
Gender	0.472	0.499	0.461	0.499	0.587	0.493	−7.932 ***
Region	2.067	0.817	2.059	0.819	2.147	0.794	−3.353 ***
Age	51.009	16.864	51.840	17.073	42.282	11.263	17.960 ***
Ethnic minority	0.075	0.264	0.076	0.264	0.071	0.257	0.547
Marital status	0.852	0.355	0.851	0.356	0.869	0.337	−1.601 *
Political appearance	0.112	0.315	0.116	0.321	0.059	0.236	5.731 ***
Education	3.230	1.511	3.196	1.525	3.567	1.291	−7.702 ***
Annual personal income	9.944	1.333	9.865	1.335	10.651	1.081	−17.792 ***
Number of minor children	0.416	0.771	0.382	0.749	0.777	0.904	−16.139 ***
Agricultural household	0.538	0.499	0.538	0.499	0.545	0.498	−0.465
Cognitive ability	2.452	0.785	2.436	0.790	2.609	0.696	−6.940 ***
Property or not	0.910	0.287	0.911	0.285	0.896	0.305	1.599
Place of residence	1.105	1.020	1.098	1.029	1.163	0.912	−2.001 **

Note: *** indicates *p* < 0.01, ** indicates *p* < 0.05, * indicates *p* < 0.1.

**Table 2:** Effect of physical exercise on entrepreneurial behavior.

Models	Probit		Tobit	
	Model 1	Model 2	Model 3	Model 4
Exercise intensity	0.008 **		0.012 ***	
	(0.003)		(0.005)	
Exercise frequency		0.028 **		0.042 **
		(0.013)		(0.020)
Gender	0.137 ***	0.138 ***	0.313 ***	0.317 ***
	(0.039)	(0.039)	(0.076)	(0.076)
Central region	0.196 ***	0.198 ***	0.054	0.052
	(0.050)	(0.050)	(0.073)	(0.073)
Eastern region	0.036	0.034	−0.037 ***	−0.037 ***
	(0.048)	(0.048)	(0.003)	(0.003)
Age	−0.023 ***	−0.023 ***	0.177	0.188 *
	(0.002)	(0.002)	(0.114)	(0.114)
Ethnic minority	0.104	0.113	0.454 ***	0.466 ***
	(0.075)	(0.074)	(0.101)	(0.101)
Marital status	0.288 ***	0.295 ***	−0.661 ***	−0.674 ***
	(0.066)	(0.066)	(0.113)	(0.112)
Political appearance	−0.435 ***	−0.444 ***	−0.094 ***	−0.098 ***
	(0.073)	(0.073)	(0.031)	(0.031)
Education	−0.060 ***	−0.063 ***	0.475 ***	0.474 ***
	(0.021)	(0.021)	(0.032)	(0.032)
Annual personal income	0.322 ***	0.321 ***	0.149 ***	0.151 ***
	(0.023)	(0.023)	(0.037)	(0.037)
Number of minor children	0.099 ***	0.101 ***	0.160 *	0.164 **
	(0.025)	(0.025)	(0.082)	(0.082)
Agricultural household	0.066	0.071	−0.235 ***	−0.239 ***
	(0.052)	(0.053)	(0.055)	(0.055)
Individual cognitive ability	−0.148 ***	−0.152 ***	0.041	0.041
	(0.036)	(0.036)	(0.101)	(0.101)
Whether there is a property	0.010	0.011	0.085 **	0.080 **
	(0.066)	(0.066)	(0.036)	(0.036)
Place of residence	−0.000	−0.002	−5.216 ***	−5.215 ***
	(0.020)	(0.020)	(0.394)	(0.393)
_cons	−3.374 ***	−3.377 ***	0.313 ***	0.317 ***
	(0.269)	(0.268)	(0.076)	(0.076)
N	9792	9824	9792	9824
Pseudo R^2^	0.138	0.139	0.110	0.110

Note: Robust standard errors are in parentheses. *** indicates *p* < 0.01, ** indicates *p* < 0.05, * indicates *p* < 0.1.

**Table 3:** Comparison of urban/rural residents.

Models	Model 5	Model 6	Model 7	Model 8	Model 9	Model 10
	Rural	Urban	Full Sample	Rural	Urban	Full Sample
EI	0.012 **	0.004	0.016 ***			
	(0.005)	(0.005)	(0.005)			
EF				0.067 ***	−0.006	0.100 ***
				(0.022)	(0.016)	(0.021)
EI × Urban			−0.013 **			
			(0.006)			
EF × Urban						−0.118 ***
						(0.025)
Controls	Yes	Yes	Yes	Yes	Yes	Yes
_cons	−3.368 ***	−3.386 ***	−3.369 ***	−3.340 ***	−3.416 ***	−3.339 ***
	(0.430)	(0.361)	(0.270)	(0.428)	(0.359)	(0.270)
N	4094	5698	9792	4102	5722	9824
Pseudo R^2^	0.142	0.148	0.140	0.145	0.148	0.143

Note: Robust standard errors are in parentheses. EI = exercise intensity, EF = exercise frequency. *** indicates *p* < 0.01, ** indicates *p* < 0.05.

**Table 4:** Comparison of different education levels.

Models	Model 11	Model 12	Model 13	Model 14	Model 15	Model 16	Model 17	Model 18
	Low	Medium	High	Full	Low	Medium	High	Full
EI	0.005	−0.010	0.019 ***	−0.009				
	(0.005)	(0.011)	(0.005)	(0.009)				
EF					0.035 **	−0.015	0.026	0.101 ***
					(0.017)	(0.027)	(0.032)	(0.032)
EI× Education				0.004 *				
				(0.002)				
EF× Education								−0.021 **
								(0.009)
Controls	Yes	Yes	Yes	Yes	Yes	Yes	Yes	Yes
_cons	−3.680 ***	−3.013 ***	−4.551 ***	−3.334 ***	−3.647 ***	−3.031 ***	−4.526 ***	−3.491 ***
	(0.365)	(0.651)	(0.671)	(0.269)	(0.365)	(0.653)	(0.666)	(0.272)
N	6039	1786	1967	9792	6057	1793	1974	9824
Pseudo R^2^	0.166	0.151	0.111	0.139	0.167	0.151	0.105	0.140

Note: Robust standard errors are in parentheses. EI = exercise intensity, EF = exercise frequency, *** indicates *p* < 0.01, ** indicates *p* < 0.05, * indicates *p* < 0.1.

**Table 5:** Comparison of different marital statuses.

Models	Model 19	Model 20	Model 21	Model 22	Model 23	Model 24
	Unmarried	Married	Full Sample	Unmarried	Married	Full Sample
EI	−0.003	0.009 ***	−0.005			
	(0.013)	(0.003)	(0.013)			
EF				0.053	0.027 *	0.042
				(0.038)	(0.014)	(0.038)
EI × Marital status			0.013			
			(0.014)			
EF× Marital status						−0.016
						(0.040)
Controls	Yes	Yes	Yes	Yes	Yes	Yes
_cons	−4.665 ***	−2.918 ***	−3.414 ***	−4.679 ***	−2.913 ***	−3.476 ***
	(0.741)	(0.301)	(0.273)	(0.743)	(0.300)	(0.280)
N	1227	8565	9792	1234	8590	9824
Pseudo R^2^	0.111	0.147	0.140	0.113	0.146	0.140

Note: Robust standard errors are in parentheses. EI = exercise intensity, EF = exercise frequency, *** indicates *p* < 0.01, * indicates *p* < 0.1.

**Table 6:** Comparison of having minor children or not.

Models	Model 25	Model 26	Model 27	Model 28	Model 29	Model 30
	No Kids	With Kids	Full Sample	No Kids	With Kids	Full Sample
EI	0.005	0.012 **	0.004			
	(0.005)	(0.005)	(0.005)			
EF				0.006	0.054 **	−0.001
				(0.017)	(0.021)	(0.016)
EI × Number of kids			0.007			
			(0.004)			
EF × Number of kids						0.049 ***
						(0.014)
Controls	Yes	Yes	Yes	Yes	Yes	Yes
_cons	−3.065 ***	−3.973 ***	−3.427 ***	−3.065 ***	−3.972 ***	−3.383 ***
	(0.358)	(0.424)	(0.272)	(0.358)	(0.422)	(0.272)
N	6921	2980	9792	6943	2991	9824
Pseudo R^2^	0.127	0.084	0.140	0.127	0.084	0.141

Note: Robust standard errors are in parentheses. EI = exercise intensity, EF = exercise frequency, *** indicates *p* < 0.01, ** indicates *p* < 0.05.

**Table 7:** Comparison of young/non-young groups.

Models	Model 31	Model 32	Model 33	Model 34	Model 35	Model 36
	Young	Non-Young	Full Sample	Young	Non-Young	Full Sample
EI	0.010 **	0.006	0.013			
	(0.005)	(0.005)	(0.011)			
EF				0.052 **	0.012	0.139 ***
				(0.021)	(0.018)	(0.040)
EI × Age			−0.000			
			(0.000)			
EF × Age						−0.002 ***
						(0.001)
Controls	Yes	Yes	Yes	Yes	Yes	Yes
_cons	−4.403 ***	−1.664 ***	−3.461 ***	−4.392 ***	−1.667 ***	−3.640 ***
	(0.398)	(0.411)	(0.273)	(0.396)	(0.411)	(0.276)
N	3257	6535	9792	3271	6553	9824
Pseudo R^2^	0.080	0.163	0.139	0.082	0.163	0.141

Note: Robust standard errors are in parentheses. EI = exercise intensity, EF = exercise frequency, *** indicates *p* < 0.01, ** indicates *p* < 0.05.

**Table 8:** Mediating effect test for the relationship between exercise intensity and entrepreneurial behavior.

Models	Positive Emotions	Entrepreneurial Behavior	BMI	Entrepreneurial Behavior	Physical and Mental Health	Entrepreneurial Behavior
	Model 37	Model 38	Model 39	Model 40	Model 41	Model 42
EI	0.006 **	0.014 ***	0.008	0.008 **	0.007 *	0.014 ***
	(0.003)	(0.005)	(0.008)	(0.003)	(0.004)	(0.005)
Positive emotions		0.002				
		(0.042)				
BMI				0.015 ***		
				(0.005)		
Physical and mental health						0.049 *
						(0.029)
Controls	Yes	Yes	Yes	Yes	Yes	Yes
_cons	1.931 ***	−3.849 ***	20.991 ***	−3.695 ***	1.014 ***	−3.927 ***
	(0.175)	(0.498)	(0.492)	(0.297)	(0.247)	(0.490)
N	3288	3283	9735	9723	3292	3287
R^2^/pseudo-R^2^	0.094	0.148	0.037	0.141	0.192	0.150
R^2^_adj.	0.090	-	0.035	-	0.188	-

Note: Robust standard errors are in parentheses. EI = exercise intensity, *** indicates *p* < 0.01, ** indicates *p* < 0.05, * indicates *p* < 0.1.

**Table 9:** Mediating effect test for the relationship between exercise frequency and entrepreneurial behavior.

Models	Positive Emotions	Entrepreneurial Behavior	BMI	Entrepreneurial Behavior	Physical and Mental Health	Entrepreneurial Behavior
	Model 43	Model 44	Model 45	Model 46	Model 47	Model 48
EF	0.033 ***	0.035	0.099 ***	0.025 *	0.068 ***	0.032
	(0.009)	(0.023)	(0.025)	(0.013)	(0.013)	(0.023)
Positive emotions		−0.002				
		(0.041)				
BMI				0.015 ***		
				(0.005)		
Physical and mental health						0.046
						(0.029)
Controls	Yes	Yes	Yes	Yes	Yes	Yes
_cons	1.954 ***	−3.843 ***	21.016 ***	−3.699 ***	1.099 ***	−3.930 ***
	(0.174)	(0.497)	(0.491)	(0.296)	(0.244)	(0.489)
N	3297	3292	9767	9755	3301	3296
R^2^/pseudo-R^2^	0.096	0.146	0.038	0.141	0.198	0.148
R^2^_adj.	0.092	-	0.037	-	0.194	-

Note: Robust standard errors are in parentheses. EF = exercise frequency, *** indicates *p* < 0.01, * indicates *p* < 0.1.

**Table 10:** Instrumental variable method.

Models	Phase I		Phase II	
	Model 49	Model 50	Model 51	Model 52
Watching sports matches	0.583 ***	0.397 ***		
	(0.099)	(0.028)		
EI			0.140 **	
			(0.060)	
EF				0.207 **
				(0.083)
Controls	Yes	Yes	Yes	Yes
_cons	−1.089 ***	−1.168 ***	−3.281 ***	−3.212 ***
	(0.670)	(0.190)	(0.271)	(0.266)
N	9780	9816	9780	9816
Wald test	-	-	5.41 ***	6.22 ***
AR test	-	-	6.28 ***	6.38 ***

Note: Robust standard errors are in parentheses. EI = exercise intensity, EF = exercise frequency, *** indicates *p* < 0.01, ** indicates *p* < 0.05.

**Table 11:** CEM, reduced sample test, and copulas test.

Models	Model 53	Model 54	Model 55	Model 56	Model 57	Model 58
EI	0.010 ***		0.009 **		0.008 *	
	(0.004)		(0.005)		(0.005)	
EF		0.033 **		0.054 **		0.055 **
		(0.015)		(0.021)		(0.024)
C_EI					−0.005	
					(0.028)	
C_EF						−0.050
						(0.040)
Controls	Yes	Yes	Yes	Yes	Yes	Yes
_cons	−3.799 ***	−3.599 ***	−4.217 ***	−4.213 ***	−3.453 ***	−3.485 ***
	(0.518)	(0.406)	(0.397)	(0.394)	(0.321)	(0.283)
N	6281	5865	3243	3257	9792	9824
Pseudo R^2^	0.131	0.129	0.080	0.080	0.140	0.140

Note: Robust standard errors are in parentheses. Bootstrap errors are in parentheses for Model 57/58. EI = exercise intensity, EF = exercise frequency, *** indicates *p* < 0.01, ** indicates *p* < 0.05, * indicates *p* < 0.1.

## Data Availability

All data included in this study are available upon request by contact with the corresponding author.
